# COVID-19 Presenting as Severe Rhabdomyolysis With Normal Renal Function

**DOI:** 10.7759/cureus.9556

**Published:** 2020-08-04

**Authors:** Akshay Shanbhag, Pritika S Manaktala, Hira Rizvi, Kevin Frey, Rama Narayanan

**Affiliations:** 1 Internal Medicine/Geriatrics, Beth Israel Deaconess Medical Center, Harvard Medical School, Boston, USA; 2 Internal Medicine, Canton Medical Education Foundation, Canton, USA

**Keywords:** covid-19, rhabdomyolysis, normal renal function

## Abstract

Coronavirus disease 2019 (COVID-19) continues to increase morbidity and mortality. Early recognition of symptoms, along with prompt intervention, is required to improve patient outcomes. COVID-19 can have a multifaceted presentation, which can be a diagnostic challenge. Here, we report the first case of COVID-19 presenting as severe rhabdomyolysis with creatine kinase > 500,000 U/L with normal renal function in a young adult.

## Introduction

Coronavirus disease 2019 (COVID-19) is currently a global health crisis caused by Severe Acute Respiratory Syndrome Coronavirus-2 (SARS-COV-2). Belonging to the Coronavirus family, which includes Middle East respiratory syndrome coronavirus (MERS) and severe acute respiratory syndrome coronavirus (SARS), a prompt diagnosis is required to improve patient outcomes. Common symptoms include fever, dyspnea, cough and fatigue, while less common symptoms are sputum production, headache and hemoptysis [1]. An atypical presentation can be a diagnostic challenge. Currently, there have been few reported cases of COVID-19 associated rhabdomyolysis in adults and few in pediatrics [2-10]. Here, we report the first case of COVID-19 presenting as severe rhabdomyolysis with creatine kinase > 500,000 U/L with normal renal function in a young adult.

## Case presentation

**Figure 1:**
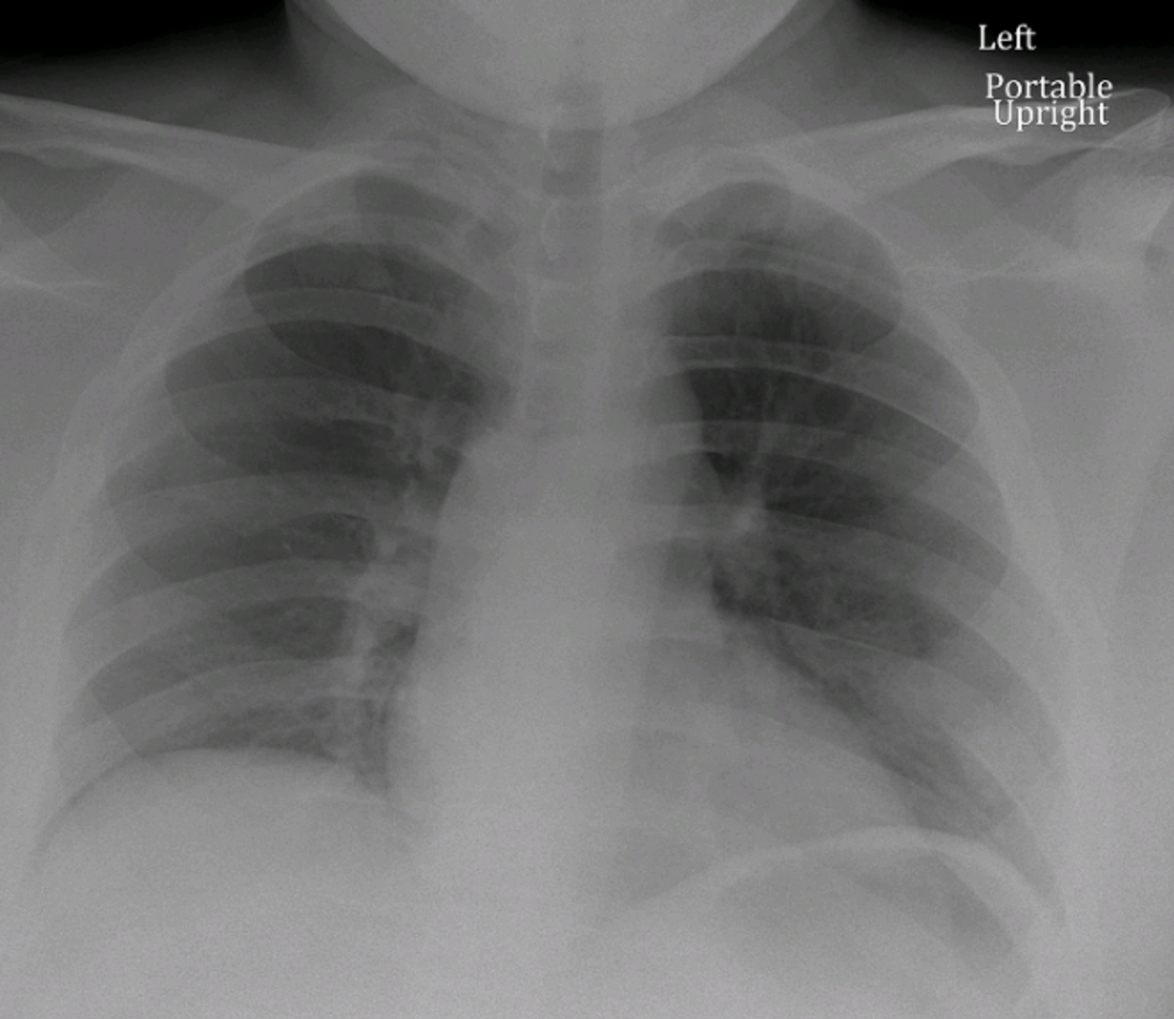
Admission chest x-ray

**Figure 2:**
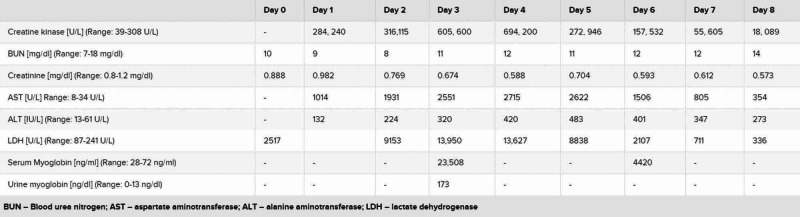
Laboratory values

A 19-year-old African-American male with a history of anxiety, prior influenza-associated rhabdomyolysis presented to the ED with a three-day history of worsening bilateral lower extremity myalgias, associated with red-colored urine and a mild dry cough. The remaining review of systems was negative. He denied exercise intolerance and family history was unremarkable for myopathies or neuromuscular disorders. Social history was negative for alcohol, smoking or illicit drug use. He endorsed working in an extended care facility with COVID-19 patients. Believing he may have COVID-19, he presented to the ED. Vitals were normal and the physical exam was remarkable only for bilateral lower extremity tenderness. Complete blood count (CBC) showed leukopenia of 2.4 x 10^9^/L (Range: 4.5-11 x 10^9^/L) with lymphocytopenia of 0.9 x 10^9^/L (range: 1-4 x 10^9^/L). Basic metabolic panel (BMP), renal function, procalcitonin, d-dimer and ferritin were normal. Serum creatine kinase (CK) and lactate dehydrogenase (LDH) were elevated at 284,240 U/L (range: 39-308 U/L) and 2517 U/L (Range: 87-241 U/L), respectively. C-reactive protein (CRP) was elevated at 0.57 mg/dL (range: 0-0.32 mg/dL). The liver panel showed aspartate aminotransferase (AST) and alanine aminotransferase (ALT) were elevated at 1014 U/L (range: 8-34 U/L) and 132 U/L (range: 13-61 U/L), respectively. Urine dipstick was positive for blood but negative for red blood cells (RBCs). Chest x-ray, respiratory viral panel, urine and serum toxicology screen were unremarkable (Figure 1). He was admitted for severe rhabdomyolysis and started on intravenous fluids. He tested positive for COVID-19 and we continued conservative management without antibiotics or hydroxychloroquine. Serum and urine myoglobin were elevated at 23,508 ng/ml (range: 28-72 ng/ml) and 173 ng/ml (range: 0-13 ng/ml), respectively. Viral hepatitis panel, aldolase, thyroid-stimulating hormone (TSH), erythrocyte sedimentation rate (ESR), anti-nuclear antibody (ANA), anti-Jo-1, anti-SSA/SSB, anti-RNP, anti-dsDNA and anti-Smith antibodies were unremarkable. His myalgias continued to worsen, now involving his upper extremities. Serum CK, LDH and AST levels continuously rose, peaking at 694,200 U/L, 13,950 U/L and 2715 U/L, respectively. His renal function and urine output, however, were within normal limits. His symptoms and levels started improving by day 5 with supportive care (Figure 2). Given his history and positive COVID-19 test, it supported COVID-19 presenting as rhabdomyolysis. His renal function, urine output and respiratory function, interestingly, did not deteriorate throughout his eight-day hospital course. During one of his prior hospitalizations for influenza-associated rhabdomyolysis, he underwent an evaluation for metabolic myopathy, which was unremarkable.

 

## Discussion

Rhabdomyolysis results from the breakdown of skeletal muscle, causing leakage of muscular contents into the circulation [11]. It is characterized by serum CK at least 10 times the upper limit of normal [12]. Clinical features occur over hours to days [13] and can range from asymptomatic CK elevation and myalgias to life-threatening conditions such as electrolyte abnormalities, cardiac arrhythmias and renal failure [12]. Inherited causes include defects in cellular metabolism while acquired causes include substance abuse, trauma, medications and seizures [12]. Influenza is one of the most common viral causes [2]. However, clinicians need to suspect inherited disorders when associated with recurrence, positive family history or exercise intolerance.

During the literature review, we found a few reported cases of COVID-19 associated rhabdomyolysis in adults [2-8]. In three of these cases, with COVID-19 associated rhabdomyolysis, the patients were above 65 years of age and also developed acute kidney injury (AKI) with elevated creatinine [3,4,6]. For the other two cases, the patients were below 50 years of age, but had an associated AKI [5,7]. There was one case of a 38-year-old male with COVID-19 associated rhabdomyolysis with normal renal function, however, his peak CK was only 42,670 U/L [8]. In pediatrics, there was a case of a 16-year-old male who presented with rhabdomyolysis, was diagnosed with COVID-19 and had a CK level over 400,000 [10]. However, he had a worsening renal function and eventually became anuric [10]. To our knowledge, there have been no cases of COVID-19 presenting with severe rhabdomyolysis with creatine kinase > 500,000 U/L with a normal renal function in a young adult. Interestingly, he also presented without the progressive pulmonary symptoms associated with COVID-19.

Our report has limitations as it lacks a confirmed etiology of the rhabdomyolysis. We did not evaluate viral causes such as HIV, Epstein Barr virus (EBV) and cytomegalovirus (CMV). Further genetic testing would be required to identify inherited disorders predisposing him to viral infection associated rhabdomyolysis. Our patient, being young and not in acute respiratory distress syndrome (ARDS), tolerated aggressive hydration. However, the treatment of rhabdomyolysis in COVID-19 patients can be challenging, as aggressive hydration can worsen respiratory function, especially if the patient has heart failure or ARDS [3].

## Conclusions

Rhabdomyolysis can be a life-threatening complication that has a high morbidity and mortality. It can present as a complication of viral infections and is also associated with COVID-19 among all age groups. Physicians need to be aware of the multifaceted presentations of COVID-19 and have rhabdomyolysis in the differential diagnosis. Treatment focuses on aggressive fluid hydration. However, this can be challenging and is limited by the patient’s respiratory status and cardiac history.
